# The Stamping Method Utilizing a Double-Trough Die in Microforming to Enhance Formability

**DOI:** 10.3390/mi15070922

**Published:** 2024-07-18

**Authors:** Ming-Hung Hsu, Kuo-Ming Huang, Chuan-Hsaing Chang, Chung-Ping Liu

**Affiliations:** 1 Department of Marine Engineering, National Taiwan Ocean University, No. 2, Beining Rd., Zhongzheng Dist., Keelung City 202301, Taiwan; jerry770630@hotmail.com (M.-H.H.); boy8805183@gmail.com (C.-H.C.); 2 Research Center of Manufacturing and Material Measurement, National Taiwan Ocean University, No. 2, Beining Rd., Zhongzheng Dist., Keelung City 202301, Taiwan; 3 Department of Merchant Marine, National Taiwan Ocean University, No. 2, Beining Rd., Zhongzheng Dist., Keelung City 202301, Taiwan; ntouimt@email.ntou.edu.tw

**Keywords:** microgear, extrusion-cutting, trough die, negative clearance, hydrostatic pressure wall

## Abstract

Currently, the field of microgear manufacturing faces various processing challenges, particularly in terms of size reduction; these challenges increase the complexity and costs of manufacturing. In this study, a technique for microgear manufacturing is aimed at reducing subsequent processing steps and enhancing material utilization. This technique involves the use of trough dies with extrusion-cutting processing, which enables workpieces to undergo forming in a negative clearance state, thus reducing subsequent processing time for micro products. We conducted finite element simulations using microgear dies, measuring stress, velocity, and flow during the forming process of four types of dies-flat, internal-trough, external-trough, and double-trough dies. The results indicated that the buffering effect of the troughs reduced the rate of increase in the material’s internal stress. In the cavity, the material experiences a significant increase in hydrostatic pressure, leading to the formation of a “hydrostatic pressure wall”. This pressure barrier imposes substantial constraints on the flow of the material during dynamic processes, making it difficult for the material to move into the remaining areas. This effectively enhances the blockage of material flow, demonstrating the critical role of hydrostatic pressure in controlling material distribution and movement. In addition, combining the characteristics of both into a double-trough die enhances the overall stability of forming velocity, reduces forming load and energy consumption, and maximizes material utilization. Results further revealed that microgears manufactured using double-trough dies exhibited defect-free surfaces, with a dimensional error of less than 5 μm and tolerances ranging from IT5 to IT6. Overall, this study offers new insights into the traditional field of microgear manufacturing, highlighting potential solutions for the challenges encountered in current microstamping processes.

## 1. Introduction

Various techniques, including traditional approaches such as milling, hot forming, gear cutting, and powder metallurgy, are used in the gear manufacturing industry. In micro-electro-mechanical processes, nontraditional techniques, such as electrical discharge machining (EDM) and wire EDM, are used in gear manufacturing. Given the intricate processes involved in the reduction of product dimensions, certain challenges pertaining to manufacturing complexity and processing precision have emerged, and thus the processing time has considerably increased. Additional challenges include the substantial investment required for the equipment needed for implementing these innovative, nontraditional techniques, particularly for the large-scale production of high-precision, small-sized gears.

In traditional gear manufacturing, powder metallurgy (PM) enables large-scale production and inherently enhances material strength. However, materials used in PM often exhibit porosity, and any variations in processing conditions can impact the mechanical properties of the final product [1]. Moreover, the high initial investment in molds hinders widespread adoption of this technology [2]. Pore formation is inevitable during PM gear manufacturing, leading to component fatigue [3]. These factors may contribute to instability in products manufactured via powder metallurgy. Subsequent rotational testing of PM gears reveals micro-pitting damage on the surface and subsurface, extending to cracks connected with subsurface voids [4]. From a user perspective, damage to any gear component reduces overall machine efficiency and may lead to occupational hazards.

Although equal channel angular pressing (ECAP) can address the sintering pore issues and achieve materials with fine-grained structures when combined with processes such as Accumulative Roll Bonding (ARB) and High-Pressure Torsion (HPT), this technique requires advanced mold technology, skilled operators, and multiple repetitive processes to achieve structural uniformity [5,6]. Therefore, it is not suitable for mass production of gears.

With current technology, both PM and ECAP processes are capable of manufacturing micro components. However, their drawbacks, including high costs and technical complexity, still prevent them from being fully integrated into mass production. Traditional stamping enables high-volume production while maintaining product precision, making it a suitable method for large-scale gear manufacturing. Cold extrusion also plays a crucial role in gear production by facilitating rapid manufacturing and ensuring product strength, thereby exhibiting excellent wear resistance during operation [7].

Metal stamping is a widely used processing technique in the field of manufacturing. In the precision blanking process of stamping, V-rings are used to increase the compressive stress of the material during the shearing process while suppressing rotational flow, thereby increasing static hydrostatic pressure [8]. In scenarios involving thick workpieces, the height limitation of V-rings reduces their applicability, making them unsuitable for processing thick workpieces.

Extrusion-cutting forming offers a novel solution for the aforementioned problem [9]. Research shows that trough dies can considerably enhance the quality of products. These dies can be combined with negative clearance technology to manufacture products with complex geometries [10,11]. The manufacturing process for trough dies is simpler and involves a lower risk of damage than that for V-rings. Trough dies can be easily fabricated on die plates or master dies. During stamping, these dies can generate considerable static hydrostatic pressure within the material, thus preventing ruptures as the material passes through the outlet. In summary, trough dies improve the processing precision and quality of the final product and are suitable for stamping thick plates.

Gears with a diameter of 0.1–10 mm are classified as microgears [12]. The researchers discovered that conducting cylindrical microforming with 1-mm gears under restricted conditions yielded optimal final products [13]. Advancements in manufacturing technologies, particularly a compound stamping technique combining forging and stamping, have opened new avenues for microgear production. Ref. [14] developed a two-stage compound forming technique involving punching and forging and thus successfully fabricated microgears with high levels of hardness. Ref. [15] reported that the burrs generated during EDM—based manufacturing of microgear molds considerably affected forming pressure and tooth integrity, necessitating further grinding treatment. Ref. [16] investigated the effect of grain size on microforming during upsetting and subsequently recommended the use of materials with large grains and appropriate clearances to reduce material deformation loads. Ref. [17] used equal channel angular pressing to conduct experiments on AL-1050 as indicated; they reported that the effect of grain misalignment on hardness was considerably stronger than that of grain size. By conducting experiments on SUS304, Ref. [18] investigated how clearances affect forming effects during the punching process. In summary, to achieve optimal manufacturing outcomes, factors such as material characteristics, process conditions, and clearance design should be carefully considered during the manufacture of microgears.

The present study seeks to evaluate the effectiveness of various extrusion-cutting forming techniques in the manufacture of microgears. We use the commercial finite element software DEFORM-3D to optimize the troughs of the die. Our objective was to identify trough configurations that induce optimal stress and flow characteristics in the material, thereby fabricating high-quality microgears. In the experimental phase, precise product measurements were performed to identify the most suitable technique for microgear processing.

## 2. Experimental and Simulation Design of Trough Dies for Microgear Forming

In this study, the forming process of microgears was investigated by comprehensively analyzing various trough designs. Corresponding experimental molds were developed to simulate optimal stress distributions and material flow outcomes. Certain practical challenges, for example, the effect of static hydrostatic pressure on material distribution, plastic flow behavior of materials, and utilization efficiency of materials, persist in the production of microgears, necessitating further in-depth exploration. These challenges are systematically discussed in the subsequent sections of this article.

Commercially pure Al-1050 workpieces with a chemical composition as indicated in Table 1 were used in the present work. The Al-1050 workpieces were machined into a cylindrical shape with a diameter of 6.5 mm and a thickness of 2 mm. Al-1050 exhibits excellent ductility and is particularly suitable for examining plastic behavior in microforming. SKD11-mold steel was selected as the target mold material because of its excellent strength, which makes it suitable for stamping various metal materials during microforming.

DEFORM-3D is a commercially available simulation software based on the finite element method, primarily used for analyzing metal forming and related industrial processes such as heat treatment. In this study, DEFORM-3D (Version: V13.1.1) was used to comprehen-sively analyze the microgear forming process. Adjustments were made to the trough values in the microgear mold depending on the characteristics of material flow. Simulations involving precise settings for plastic flow stress and friction coefficients were performed, and key parameters such as stamping speed, stroke, and die pressure were carefully configured. It is noteworthy that the mold operates by first applying pressure to the material before proceeding with extrusion-cutting; hence, the blank holding force is set as a constant rather than a dynamic. The friction coefficient is the value obtained from the ring compression test using R68 + MoS2 as the medium. (Refer to Table 2 for detailed information). To determine the effects of microforming, a nine-tooth gear was selected for experimentation. Table 3 presents the gear design and simulation parameters in detail.

**Figure 1:**
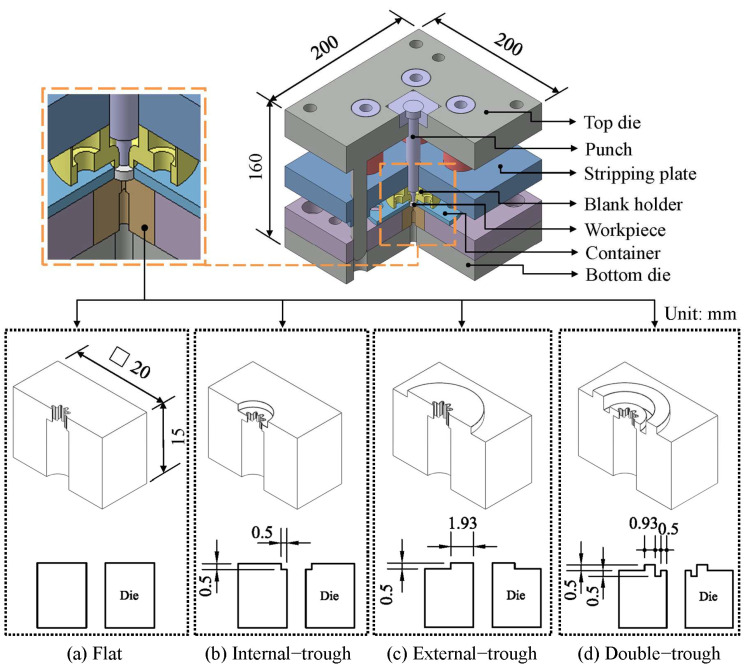
Four types of microgear die designs and the dimensions of the micro features.

Variations in the geometric shapes of dies may induce changes in static hydrostatic pressure during the forming process, thus affecting the stability of material flow and ultimately leading to variations in the quality of the final product. In this study, the effects of different die trough forms were investigated by examining dies with four distinct geometric features: flat, internal-trough, external-trough, and double-trough (Figure 1).

To explore the impacts of trough design and extrusion-cutting techniques on microforming, this study utilized a flat die without troughs as the control benchmark. In the context of stamping small objects, achieving near-final dimensions in the base material is essential to minimize material waste and mitigate die damage caused by excessive extrusion pressure. As dimensions decrease, there is a concurrent escalation in initial material processing costs and manufacturing complexity. Addressing these challenges, an internal-trough die was developed based on the extrusion ratio principles inherent to extrusion dies.

Regarding external-trough dies, the study considers the extrusion-cutting techniques where, in addition to achieving effective extrusion, maintaining appropriate hydrostatic pressure within the material is essential for optimal shearing actions. Given the demonstrated benefits of trough designs in enhancing product quality during stamping and shearing processes [19], this study integrates the use of trough dies into the research on microgear formation and defines these as external-trough dies.

**Figure 2:**
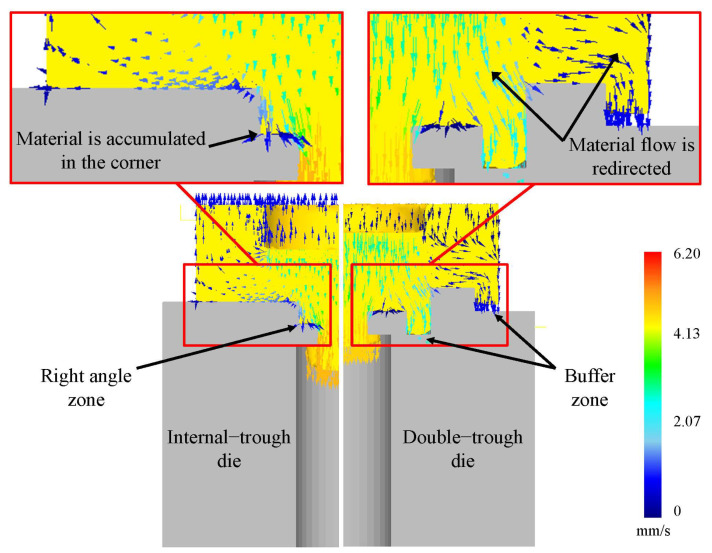
Material flow patterns in internal-trough and double-trough dies.

The design of the double-trough involves combining internal and external dies, followed by further improvement. The material is known to undergo compression when passing through the right-angle zone of the internal-trough, leading to material hardening or uneven flow. The double-trough design is adopted for the most appropriate plastic deformation, and it is a new die pattern obtained by combining the internal and external-trough dies. Therefore, as shown in Figure 2, we added a trough within the internal trough to facilitate continuous deformation flow in the right-angle zone during material bending. These dies were comprehensively analyzed to evaluate their effects on the forming.

## 3. Numerical Analysis Simulation of Trough Dies

During material deformation, changes in the geometric shapes of dies directly affect the outcomes of forming. Adjusting the die geometry may alter the material’s internal stress and flow patterns. Therefore, changes in the geometry of the die will be the focus of this simulation analysis. In this study, the precise measurements of stress and velocity focused on three key locations: the center, dedendum, and addendum of the ejection port. Various aspects of material flow, load conditions, and stamping energy were comprehensively examined under different mold-forming conditions. Finally, the optimal mold was subjected to a validation analysis to obtain deeper insights into the process.

### 3.1. Stress Analysis

**Figure 3:**
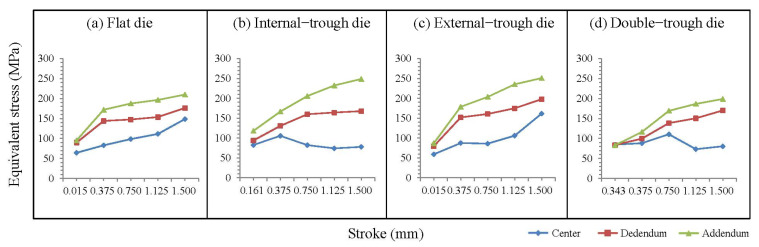
Illustration of stress curve under microgear forming.

Figure 3a shows three stress curves for forming with a flat die. The developmental trends of these three curves indicated that the flat die failed to provide an adequate buffering zone for the material. Consequently, with the progression of stroke, an increasing trend is observed in stress values at the three locations, with the most noticeable stress growth occurring in the central region.

Figure 3b depicts the stress curves for forming with an internal-trough die. The growth pattern of the stress curve for the addendum is similar to that of the external-trough die. However, a distinct pattern is observed in the central stress curve, attributable to the buffering effect of the internal trough. The central stress considerably decreases when the stroke exceeds 25%, and this decrease continues until the end of the stamping process. The curve for the dedendum tends to stabilize after the stroke exceeds 50%. This trend can be explained by the internal-trough structure providing additional buffering space at the material center, effectively reducing and inhibiting stress growth at the material center.

Figure 3c shows the stress curves for forming with an external-trough die. The growth pattern of the curves at the dedendum and addendum is similar to that of the flat die because the ejection port position is the same as that of the flat die. Nevertheless, because of the protrusion of the external-trough edges, the static hydrostatic pressure within the material increases, resulting in final stress values exceeding those generated by the flat die.

Figure 3d depicts the stress curves for forming with a double-trough die. The stress values observed at the center, dedendum, and addendum are lower than those observed with the other die forms. Before the stroke reaches 50%, the buffering effect of the internal trough reduces the overall rate of increase in material stress, although the stress in the central region slowly increases. However, when the stroke exceeds 50%, the material comes into full contact with the bottom of the internal trough, causing the buffering zones generated by the external and internal troughs to overlap. Consequently, the stress shifts from the central region to the external trough, leading to a substantial decrease in the central stress of the material.

From the aforementioned results, the following conclusions can be drawn:

(1) Because of the lack of material buffering space, internal stress increases with the progression of the stroke;
(2) Introducing an internal trough generates a buffering effect in the intermediate stages of stamping and suppresses the increase in stress with the progression of the stroke;
(3) Introducing an external trough generates a buffering effect in the early stages of forming and suppresses the increase in stress throughout the forming process;
(4) Double-trough dies combine the characteristics of both external and internal troughs, suppressing the increase in stress in the early stages of stamping and further suppressing the increase in central stress in the intermediate stages.

### 3.2. Velocity Analysis

**Figure 4:**
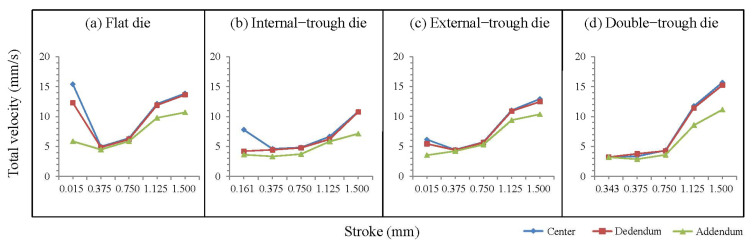
Illustration of velocity curve under microgear forming.

Figure 4a shows the velocity curve obtained when a flat die is used. According to the growth pattern of the curve, the velocities observed at the center, dedendum, and addendum are significantly higher than those of dies designed with a trough form. High velocities are observed, particularly at the center and dedendum. This phenomenon presumably occurs because of the lack of a buffering effect provided by troughs during the material’s passage through the ejection port. This lack of troughs enables the material to preferentially flow toward regions with low resistance after undergoing compression, thereby inducing high flow velocities in the central and dedendum regions.

Figure 4b depicts the velocity curve obtained when an internal-trough die is used. Designing an internal trough involves a two-stage forming process, during which the material is formed into a cylinder in the early stages of stamping. Initial observation of the curve reveals a higher velocity at the center than at the periphery. As the material passes through the ejection port, the velocity observed at the dedendum considerably decreases because of the reduced volume of material during the first stage of forming, which limits the flow rate at the dedendum. By contrast, the internal trough restricts the overall influx of material into the ejection port, resulting in a low flow rate at the addendum during the later stages of forming, thereby leading to variations in velocity between the center, dedendum, and addendum.

Figure 4c shows the velocity curve obtained when an external-trough die is used. This design features a trough form on the outer side of the die, resulting in a prominent buffering effect during the early stages of stamping. The presence of a trough substantially reduces the velocity of flow at the central and dedendum positions. In addition, the gear geometry results in variations in velocity at the beginning of the stamping process. Overall, the curve indicates a slightly lower velocity at the addendum compared with the central and dedendum velocities, presumably because the material achieves plasticity after passing through the ejection port. During the subsequent growth stage of the final product, while the material passes through the ejection port, it maintains its original velocity, ultimately resulting in a major protrusion at the center of the final product. With the progression of stamping, when the stroke reaches 50%, the material comes into contact with the troughs, gradually diminishing the buffering effect. Eventually, all the velocities of the material substantially increase until the end of the stamping process.

Figure 4d depicts the velocity curve obtained when a double-trough die is used. Initial observation of the growth pattern of the curve reveals a major change after the stroke exceeds 50%. At this point, the material comes into contact with the bottom of the internal trough, leading to a reduction in the buffering effect and a subsequent rapid increase in central velocity. Before the stroke reaches 50%, the curves of the center and dedendum almost overlap, and the velocity at the addendum does not decrease until after the stroke reaches 50%. In addition, the curve of the addendum continues to follow the curves of the center and dedendum. This phenomenon underscores the substantial effect of double-trough molds on the microgear forming process, particularly after the stroke exceeds 50%. Among the velocity curves for all molds, only those obtained with a double-trough mold exhibit similar variations in the curves for the center, dedendum, and addendum. These findings indicate that the design of double-trough dies effectively minimizes the variations in velocity across material regions during gear forming.

According to the analysis of velocity curves, the design of troughs provides a buffering space for the material as it passes through the ejection port. This design minimizes the variations in velocity at the center, dedendum, and addendum. The smaller these variations are, the higher the consistency in the material curves is during the forming process, indicating the role of trough design in achieving a uniform velocity distribution. Overall, this feature underscores the key role of trough design in microforming. By providing a suitable buffering zone, the mold ensures that the material experiences a uniform velocity at the ejection port, which consequently affects the overall flow characteristics of the forming process.

### 3.3. Forming Load and Energy

**Figure 5:**
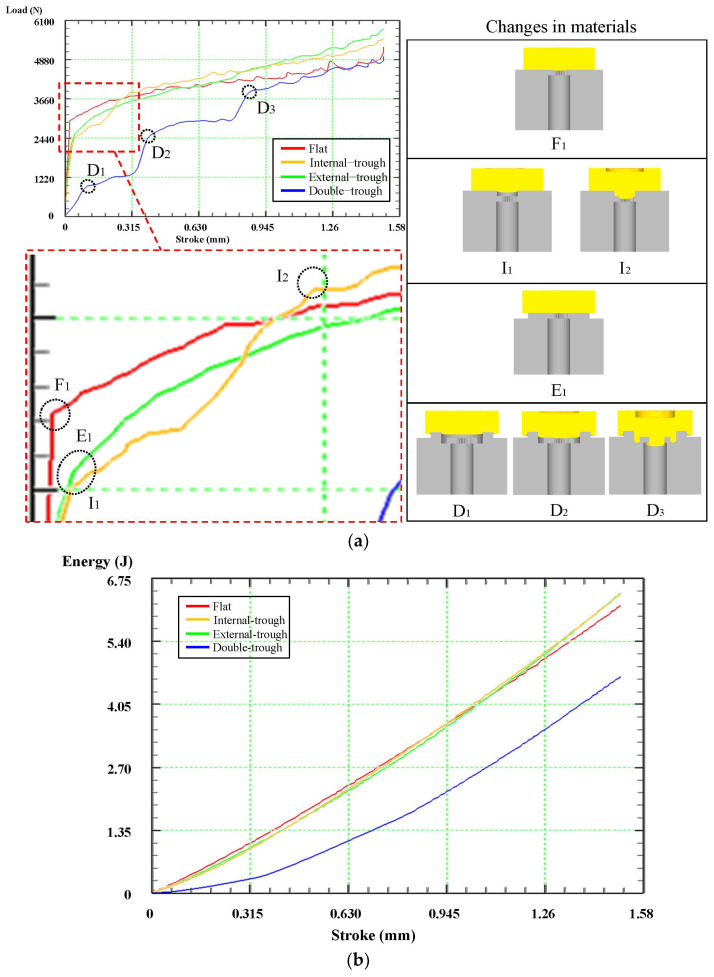
The punch curve diagram for forming microgears with trough dies. (**a**) Load curve of microgear forming. (**b**) Total energy consumption curve for microgear forming.

During the process of stamping, the load borne by the punch is influenced not only by the stroke but also by variations in die design. In this study, variations in load and energy caused by trough dies during the forming process were examined. As shown in Figure 5a, when forming was performed using a flat die, the punch load rapidly increased and reached its maximum after the initiation of forming and remained high until the end of stamping. By contrast, when a trough die was used, a distinct growth pattern was observed in the load curve, and a major reduction was observed in the punch load during the forming process. This growth pattern reflected the mode of energy distribution in the trough die during the forming process.

During an in-depth investigation of trough design, initial focus was on the load curves for external-trough dies. It was observed that, during the initial stages, the increase of load was slower for external-trough dies compared to flat dies.

A stair-step pattern was observed in the load curve of the internal-trough die, presumably resulting from material filling into the internal trough during the initial stages of forming. In this phase, the large cavity area of the die facilitated the process of material flow, thus reducing the initial load of the punch. Nevertheless, with the stroke progression, the material started to flow through a smaller outlet, and the punch load increased.

Overall, the curves for the double-trough die depicted three stages of load growth. The first increase in load occurs after stamping when the material fills the internal trough. Because the size of the die cavity decreases, the flow of the material is constrained, increasing the punch load. The second increase in load occurs as the material enters the ejection port, influenced by the reduction in flow area, resulting in another increase in the punch load. The third increase in load occurs when the material comes into contact with the bottom of the internal trough, diminishing the buffering effect of the internal trough and causing another increase in the punch load. Although only the external trough continues to play a role at this moment, as observed from the curve, the double-trough configuration minimizes the punch load, thus extending the lifespan of both the punch and the die.

As indicated by the stamping-energy curve depicted in Figure 5b, since the displayed curve represents the total energy generated by various dies during the forming process, this segment does not show peaks corresponding to die load. In this figure, each type of die generates a unique punch load during the stamping process, leading to variations in the required stamping energy. Graph analysis revealed similar energy curves for dies, except for double-trough dies, during the stamping process. Therefore, utilizing a double-trough die to form microgears not only diminishes the load on the punch and die but also provides the pressing machine with increased flexibility in tonnage selection.

### 3.4. Material Flow Analysis

**Figure 6:**
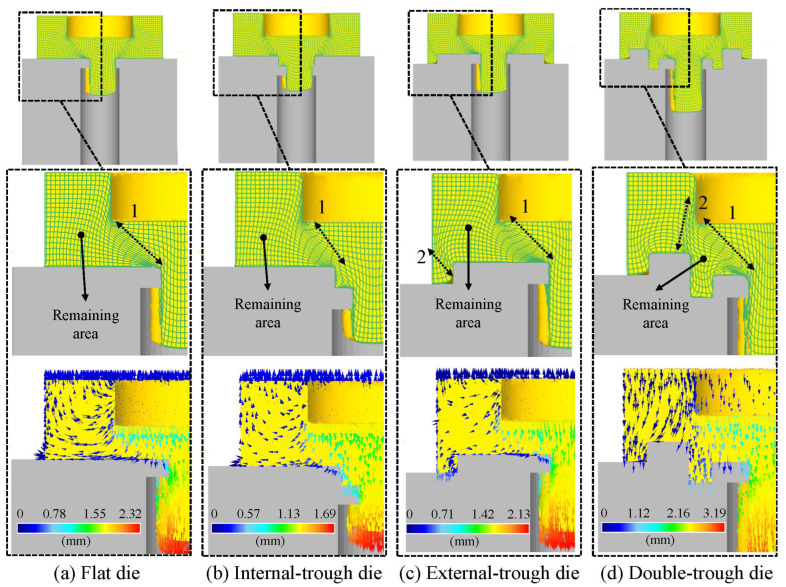
Mesh and element displacement states at 75% tool stroke with static hydrostatic pressure wall quantities (1 and 2).

**Figure 7:**
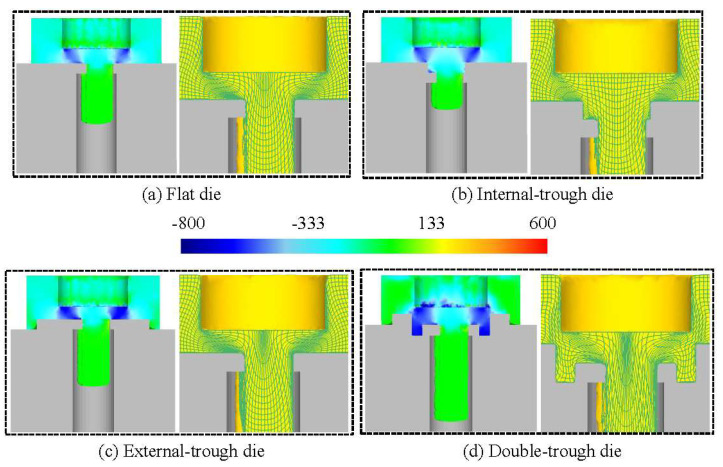
The mesh and the mean stress distribution at 100% of the tool stroke.

Material flow patterns are influenced by die geometry, resulting in different curve formations. The most critical changes occur in the final stage of the tool stroke. Figure 6 and Figure 7 depict simulated material flow patterns for flat, internal-trough, external-trough, and double-trough dies, with a tool stroke ranging from 75% to 100%. It is noteworthy that the maximum stress–strain regions in the material deformation process are predominantly located between the die and punch edges of the die. This area, characterized by dense mesh, creates a barrier known as the “hydrostatic pressure wall,” which impedes the direction of material flow. Variations in the material flow pattern for each type of trough within this stroke range are discussed below.

(1) Flat die

In Figure 6, when a flat die is used at a stroke of 75%, dense meshes are observed from the ejection port to the punch-cutting edge. These concentrated meshes form a wall (marked as 1 in Figure 6a), creating a distinct boundary in the material displacement. This indicates that the effect of this barrier can obstruct the material flow toward the remaining areas. However, the results of Figure 7a illustrate that when the stroke is increased to 100%, a single hydrostatic pressure wall could not effectively prevent the material from flowing into the remaining area, resulting in relatively short stamped products.

(2) Internal-trough die

When an internal-trough die is used at a stroke of 75%, the internal-trough structure demonstrates the same grid effect as the flat die (Figure 6b). Once again, it is demonstrated that a single hydrostatic pressure wall cannot effectively prevent the flow of material into the remaining area. Furthermore, due to the influence of the internal-trough structure, there is more space occupied by the material before passing through the ejection port compared to the flat die. Although the excess space helps to slow down the material’s passage through the ejection port, it also reduces the amount of material passing through, resulting in shorter product lengths. As shown in Figure 7b, this situation will become even more apparent when the tool stroke reaches 100%.

(3) External-trough die

When an external-trough die is used at a stroke exceeding 75%, in addition to the dense meshes displayed from the punch to the ejection port, dense meshes also appear near the edge of the external-trough, creating two hydrostatic pressure walls that hinder material flow. Given the geometry of external troughs, a slight deviation is observed in the mesh of the material’s edges, resulting in major differences compared with those observed for flat dies.

Similar to flat dies, the external-trough dies have the first hydrostatic pressure wall extending to the ejection port (marked as 1 in Figure 6c). However, the deformation of the grid at the edge of the external-trough creates a second hydrostatic pressure wall for the material (marked as 2 in Figure 6c). The area enclosed by hydrostatic pressure walls 1 and 2 forms a new remaining area, which slightly reduces the volume of the remaining area compared to the previous two dies. This allows more material to flow toward the ejection port. Despite the increased material flow toward the nozzle under the obstruction of the two hydrostatic pressure walls, the remaining area is still considerably large.

As shown in Figure 7c, when the stroke reaches 100%, the mesh density of the two hydrostatic pressure walls is further increased. At this point, the central area of the material shows the extent of mesh deformation through the ejection port, showing that the curvature of the mesh lines is greater than that of the flat die and the internal-trough die; this also indicates that the material in the central area is moving significantly. The results show that the length of the product through the ejection port is greater than that of the flat die and the internal-trough die.

(4) Double-trough die

When the tool stroke of the double-trough die is at 75%, the same situation as with the external-trough die occurs, where the material forms two hydrostatic pressure walls along the edges of the trough and the punch (marked as 1 and 2 in Figure 6d). It is worth noting that the area surrounded by these two hydrostatic pressure walls is smaller than that of the external-trough die, indicating that using the double-trough die can produce the minimum amount of remaining area, which will help maximize material utilization.

As shown in Figure 7d, when the tool stroke reaches 100%, the lines of the central mesh exhibit significant deformation, indicating that under the influence of both hydrostatic pressure walls, the majority of the material moves toward the ejection port, ultimately producing the longest finished product among all dies.

According to our aforementioned analysis, the position of the trough exerts a major effect on the variations of the mesh. As indicated by the average stress distribution depicted in Figure 7, the wall formed by dense mesh affects the coverage area of hydrostatic pressure, with the double-trough design exhibiting optimal effectiveness. Specifically, the hydrostatic pressure wall resulting from trough positions increases the degree of resistance to outward material flow. The process of material flow is hindered by hydrostatic pressure, and the material is forced to flow toward the center.

### 3.5. The Impact of Trough Design on Material Utilization

In the context of forming small parts through stamping, the variation in material volume distribution serves as a crucial indicator of plastic flow. Given the stringent requirements of microforming techniques on product dimensions and shape precision, optimal geometric design of trough dies is essential for achieving high-quality precision in small-scale components. However, this design approach is often accompanied by the drawback of lower material utilization efficiency. Continuous process optimization and improved production management practices can mitigate this limitation to some extent.

The location of the hydrostatic pressure wall is related to the geometric design of the trough, as depicted in Figure 6a–d. Slow material flow generates a hydrostatic pressure wall against fast material flow, which can block excessive material flow into dead zones to enhance material utilization efficiency.

The material utilization rates for manufacturing microgears using four types of trough dies are presented in Table 4. Considering that the material maintains constant mass during compression, calculations will utilize the initial volume of the raw material rather than the total volume after compression. The calculation formula is as follows:

$$\mathrm{Material}\;\mathrm{utilization}\;\mathrm{rate}=\frac{\mathrm{Volume}\;\mathrm{of}\;\mathrm{microgear}\;\mathrm{product}}{\mathrm{Volume}\;\mathrm{of}\;\mathrm{raw}\;\mathrm{material}}\times 100\%$$

The results indicate that microgears formed using internal-trough dies exhibit the smallest finished product volume and the largest remaining material volume, resulting in an overall material utilization rate of only 3.56%. This outcome is attributed to the initial stage of forming with the internal-trough die, where the material is initially compacted into smaller areas within the troughs to reduce stamping loads, resulting in an initial loss in the volume of the microgear product. Furthermore, in the later stages of the forming simulation, the effectiveness of the internal-troughs in creating a single hydrostatic pressure wall within the material proves insufficient to completely block the flow of some material toward the remaining areas, resulting in a reduced amount of material passing through the ejection port.

The flat die and external-trough die do not incorporate excess space before the ejection port, resulting in material utilization rates increasing to 6.13% and 6.54%. However, due to the significant remaining material areas in both types of dies, the material continues to flow toward these regions. Therefore, even with an increase in product length, further optimization can enhance the quantity of material passing through the ejection port.

Using double-trough dies for forming, the optimal material utilization rate achieved is 9.13%. The material benefits from the characteristics of both internal and external troughs, creating two hydrostatic pressure walls and minimizing residual areas. This effectively blocks more material from flowing toward those areas, resulting in the longest finished product and the least residual material volume. This design feature explains why a double-trough die ensures the optimal length of stamped products.

## 4. Measurement of Experimental Finished Product Using a Double-Trough Die

In this study, we discovered that double-trough dies outperform other dies in terms of material stress and velocity. Further examination of the simulation results of material flow revealed that the hydrostatic pressure walls formed by double-trough dies can optimize the utilization of materials. Therefore, we ultimately selected a double-trough die design for experimentation and measurement.

**Figure 8:**
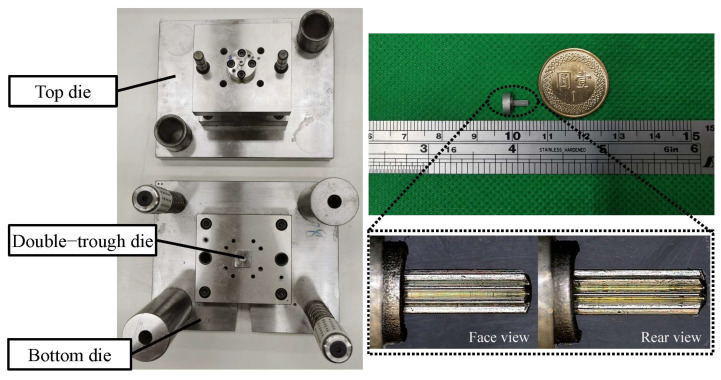
Double-trough die of finished sample.

**Figure 9:**
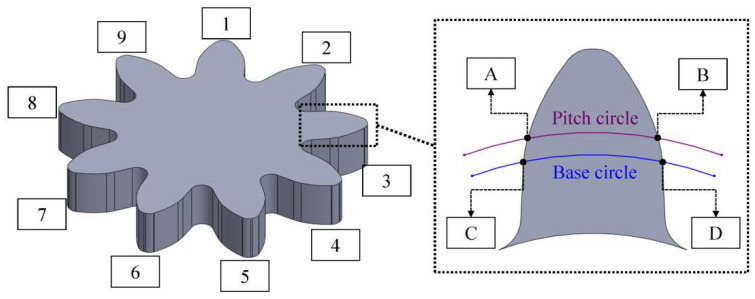
Measurement method of the experimental product. Measurement methods for experimental products (corresponding to Table 6).

Figure 8 presents the experimental results of our die and microgear, for which we utilized an optical imaging measuring instrument (Model: EVM-1510, Resson Technologies Co. Ltd., New Taipei City, Taiwan) for the measurement of the microgear (experimental results). The microgear had a length of 5.022 mm and was free of defects. We further compared tooth shape between the microgear and the original design (Figure 9). After measurement, the deviation range was within 5 μm for the dimension (Table 5 and Table 6). The experimental results met the tolerance levels of IT5. These findings further confirm the effectiveness of double-trough dies in the manufacturing of microgears.

## 5. Conclusions

In this study, our objective was to enhance the formability of materials. We used a finite element analysis technique to design four dies with different geometric shapes. To ensure optimal material stress and flow effects, we conducted mold-opening experiments to validate the results of our analysis. Ultimately, we developed an efficient stamping technique for producing high-precision microgears. The main conclusions of this study are as follows:

(1) Trough dies provide a buffering space for materials, suppress the increase in internal stress, and direct the material flow toward the ejection port by establishing hydrostatic pressure walls;
(2) In terms of trough design, the design of internal troughs effectively limits the amount of material passing through the ejection port, thereby preventing uneven flow during the early stages of forming. The design of external troughs provides a material buffer zone, which helps ensure stable material flow during the later stages of forming. The design of double troughs combines the characteristics of both internal and external troughs, and it aids in maintaining a consistent material flow rate and achieving a uniform forming process;
(3) The double-trough die induces three stages of load growth during the stamping process, reducing the load on the die and effectively decreasing the energy required for forming;
(4) The length of gears formed by double-trough dies is 2.5 times the thickness of the original blank. A large amount of the material is successfully directed toward the outlet, thus achieving effective material utilization;
(5) In experiments involving double-trough dies, optical measurements of the produced microgears reveal an average deviation of <5 μm for each dimension, with tolerance levels ranging from IT5.

## Figures and Tables

**Table 1:** Chemical composition of commercially pure aluminum Al-1050 (mass fraction, %).

Si	Fe	Cu	Mn	Mg	Zn	Ti	Al	Others
0.25	0.40	0.05	0.05	0.05	0.05	0.03	99.50	0.03

**Table 2:** FEM simulation setup.

Project	Parameter
Workpiece type	Al-1050 (plastic)
Tool type	SKD11 (rigid)
Calculation type	Lagrangian incremental
Solver	Sparse
Iteration method	Direct iteration
Flow stress function	$\bar{\sigma }=c{\bar{\varepsilon }}^{n}{\dot{\bar{\varepsilon }}}^{m}+y$
Yield function type	Von Mises
Number of elements	130,000
Constant shear friction	0.2395
Tool velocity (mm/s)	3
Blanking-holder force (N)	3400

**Table 3:** Microgear design parameters.

Project	Parameter
Gear teeth	9
Modulus	0.15
Pitch diameter (mm)	1.35
Base diameter (mm)	1.27
Tip diameter (mm)	1.65
Root diameter (mm)	0.988

**Table 4:** Simulated material volume after forming.

Die Type	Microgear Volume (mm^3^)	Remaining Area Volume (mm^3^)	Material Utilization (%)
Flat	4.07	61.24	6.13
Internal-trough	2.36	62.69	3.56
External-trough	4.34	60.82	6.54
Double-trough	6.06	57.87	9.13

**Table 5:** Comparison of experimental results and errors.

Project	Standard	Experiment	Error (μm)
Tip diameter (mm)	1.65	1.646	4
Root diameter (mm)	0.988	0.984	4
Chordal thickness (mm)	0.234	0.235	1
Length (mm)	—	5.022	—
Surface roughness (Ra)	—	0.098–0.21	—
Burrs	—	None	—

**Table 6:** Measurement values of the experimental product (unit: μm).

Project	A	B	C	D	Average
1	1.0	2.4	2.9	3.1	2.4
2	1.8	3.6	4.1	0.8	2.6
3	0.7	1.9	9.2	2.3	3.5
4	4.4	2.2	8.2	2	4.2
5	3.0	0.6	1.0	0.5	1.3
6	0.1	0.8	0.1	1.3	0.6
7	1.8	2.2	0.7	5.1	2.5
8	0.3	8.9	0.9	1.0	5.0
9	0.2	1.7	0.7	2.5	1.3

## Data Availability

Data are contained within the article.
